# Efficacy of Myofascial Trigger Point Dry Needling in the Prevention of Pain after Total Knee Arthroplasty: A Randomized, Double-Blinded, Placebo-Controlled Trial

**DOI:** 10.1155/2013/694941

**Published:** 2013-03-27

**Authors:** Orlando Mayoral, Isabel Salvat, María Teresa Martín, Stella Martín, Jesús Santiago, José Cotarelo, Constantino Rodríguez

**Affiliations:** ^1^Physical Therapy Unit, Hospital Provincial de Toledo, Cerro de San Servando s/n, 45006 Toledo, Spain; ^2^Unit of Physiotherapy, Department of Medicine and Surgery, Faculty of Medicine and Health Sciences, Rovira i Virgili University, Carrer Sant Llorenç 21, 43201 Reus, Spain; ^3^Orthopedic Surgery Service, Hospital Provincial de Toledo, Cerro de San Servando s/n, 45006 Toledo, Spain

## Abstract

The aim of this study was to determine whether the dry needling of myofascial trigger points (MTrPs) is superior to placebo in the prevention of pain after total knee arthroplasty. Forty subjects were randomised to a true dry needling group (T) or to a sham group (S). All were examined for MTrPs by an experienced physical therapist 4–5 hours before surgery. Immediately following anesthesiology and before surgery started, subjects in the T group were dry needled in all previously diagnosed MTrPs, while the S group received no treatment in their MTrPs. Subjects were blinded to group allocation as well as the examiner in presurgical and follow-up examinations performed 1, 3, and 6 months after arthroplasty. Subjects in the T group had less pain after intervention, with statistically significant differences in the variation rate of the visual analogue scale (VAS) measurements 1 month after intervention and in the need for immediate postsurgery analgesics. Differences were not significant at 3- and 6-month follow-up examinations. In conclusion, a single dry needling treatment of MTrP under anaesthesia reduced pain in the first month after knee arthroplasty, when pain was the most severe. Results show a superiority of dry needling versus placebo. An interesting novel placebo methodology for dry needling, with a real blinding procedure, is presented.

## 1. Introduction

Myofascial pain syndrome (MPS) is a highly prevalent pain condition [1, 2] caused by myofascial trigger points (MTrPs), identifiable as highly localized and hyperirritable spots in palpable taut bands of skeletal muscle fibers [3]. Among many other techniques, dry needling is frequently employed to treat MTrPs [4, 5].

Steinbrocker is usually quoted as the first to describe the effectiveness of punctures without the injection for pain management [6]. Since then, there have been numerous studies showing the effectiveness of dry needling. Some have shown that dry needling is as effective as the injection of various substances for the treatment of MTrPs [7–10].

All available reviews about the effectiveness of dry needling [11–13] reached the conclusion that dry needling appears to be an effective treatment, although studies are needed to elucidate whether its effects are superior to placebo [11, 13]. Due to the invasive nature of dry needling, it is rather difficult to design double-blinded, placebo-controlled studies to analyse its effectiveness [4, 5]. Placebo needles [14, 15] or other sham needling procedures [16] are questioned because they involve some kind of physiological stimulation, which disqualifies them as true placebo interventions [4, 17–19]. In addition, blinding with sham needling is highly dependent on the correct selection of the subjects, whom should be naive to the procedure [15, 16], and on the ability of the clinician performing the procedure [20] giving rise to as much as 20% of subjects unblinded beyond chance.

Some studies claimed to have used a double-blinded, placebo-controlled methodology with placebo needles for the sham needling group, but they elicited local twitch responses in the intervention group without actually assessing the blinding procedure [21]. A local twitch response (LTR) is a brief involuntary contraction of the fibres of the taut band that harbour the MTrP. For a study to be considered double blinded, subjects in all groups of the study must be blinded to group allocation. Since LTRs are unequivocally felt by most patients, it is hard to understand that subjects in the intervention group were really blinded, which adds to the aforementioned blinding limitations of placebo needles. 

To avoid these biases, we conducted a randomized, double-blinded, placebo-controlled clinical trial about the effectiveness of MTrPs dry needling in the prevention of myofascial pain after total knee replacement, using a novel blinding methodology.

Total knee arthroplasty has shown to be an effective treatment for knee pain due to knee osteoarthritis, providing patients with improvements in function and in quality of life with low complication rates [22]. It has been reported, however, that in the first month after surgery almost half of the patients have significant pain (>40 in visual analogue scale) [23].

MTrPs are common in lower limb muscles in patients with hip and/or knee osteoarthritis [24], and several papers have emphasized the importance of treating these MTrPs to relieve pain in osteoarthritis of both joints [24–26].

The aim of this study was to find out whether dry needling of MTrPs is superior to placebo in the prevention of pain after total knee arthroplasty, using a novel blinding methodology.

## 2. Material and Methods

### 2.1. Research Design

The study was designed as a randomized, double-blinded, placebo-controlled clinical trial. The Ethical and Clinical Research Committee of *Complejo Hospitalario de Toledo* (Spain) approved the study protocol. All subjects were interviewed individually to provide them with details about the nature of the study. All subjects voluntarily signed consent forms prior to entering the study.

### 2.2. Subjects

**Figure 1:**
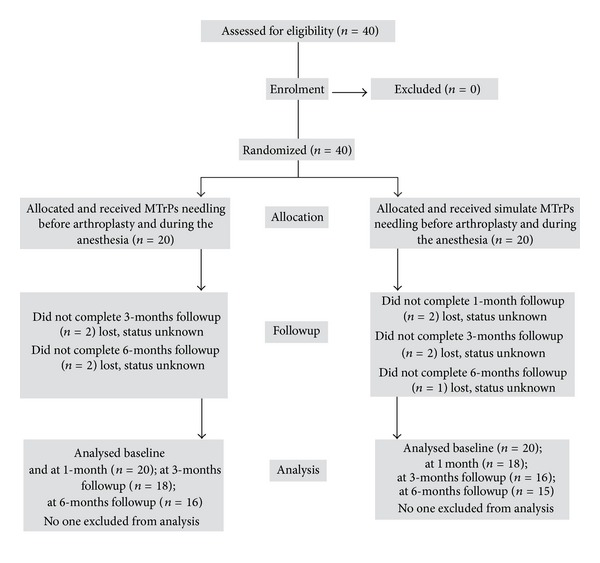
Progress of participants through the study.

Forty subjects were recruited between January 2007 and April 2008. To be included in the study, all subjects had to fulfil these criteria: (1) diagnosis of knee osteoarthritis and scheduled for total knee replacement surgery; (2) presence of active or latent MTrPs in at least one of the muscles included in the examination protocol. Patients were excluded from the study if they (1) suffered from any other condition that could cause myofascial or neuropathic pain in the lower limb, such as lumbar radiculopathy, saphenous nerve entrapment, or meralgia paresthetica; (2) presented any condition usually considered a perpetuating factor of MTrPs, such as fibromyalgia, hypothyroidism, or iron deficiencies [27]. There were no subjects who were excluded based on the study criteria (Figure 1).

### 2.3. Intervention Description

The study was carried out between January 2007 and October 2008. An experienced and trained physical therapist, blinded to the group allocation, examined the subjects several hours before surgery and at months 1, 3, and 6 after surgery. 

Subjects were assigned to a true dry needling group (T) or to a sham dry needling group (S) by using a computerized randomization list (Epidat software program, Xunta de Galicia, Spain).

Immediately after each subject was anesthetized and right before surgery started, a trained and experienced physical therapist applied dry needling to all MTrPs previously identified in the T group, using Hong's fast-in, fast-out technique [3, 28] with 0,30 × 50 mm solid filament needles. The number of insertions of the needle in each MTrP was 20, and the patient position in which every MTrP was needled was the same as the position employed by the blinded examiner for diagnosis (Table 1) and marking of MTrPs. For those MTrPs in the gastrocnemius muscles that were located right behind the knee, dry needling was not applied to avoid injuries in tibial or peroneal nerves. Subjects in the S group did not receive any treatment for their MTrPs. For subjects under spinal anaesthesia in either group (25% in the T group and 35% in the S group), a screen was used in order to prevent the patient from seeing his/her lower limbs. In subjects under spinal anaesthesia in the S group, the physical therapist simulated the application of dry needling without actually applying any treatment. Since subjects could neither see nor feel anything, they were completely blinded to group allocation. Obviously, the physical therapist applying needling was not blinded to group allocation but he did not participate in the data analysis.

### 2.4. Main Outcomes

The pain visual analogue scale (VAS) [29] was the primary outcome measure. The secondary outcomes measures were the postoperative demand for analgesics, the presence of active or latent MTrPs, the prevalence of MPS, and the Western Ontario and McMaster Universities Osteoarthritis Index questionnaire (WOMAC) [30]. Range of motion (ROM) of the knee and peak isometric strength of knee flexors and extensors was also assessed using a digital inclinometer (12-1507 Baseline, Fabrication Enterprises, Inc., NY, USA) and a digital dynamometer (Microfet 2, Hoggan Health Industries, Salt Lake City, UT, USA) respectively. 

During all checkpoints (at months 1, 3, and 6 after surgery), subjects were assessed using all these outcome measures, except for the use of analgesic.

The VAS consisted of a 100 mm line with the endpoints “no pain” and “worst pain imaginable”. 

Two days after surgery, the use of analgesic medications was recorded for a period of 4 days. Note that during the first two days, all subjects received intravenously applied analgesics consistent with the hospital's standard protocol.

Several hours before surgery, subjects were examined by an experienced physical therapist for the presence of active or latent MTrPs in the muscles of the involved lower extremity using the criteria described by Simons et al. [3]. The tensor fasciae latae, hip adductors, hamstrings, quadriceps, gastrocnemius, and popliteus muscles were examined in each subject as these muscles are frequently involved in myofascial knee pain. The examination of MTrPs followed a strict protocol regarding patient and limb positions (Table 1), the manual examination of each muscle and the marking of the MTrPs with a blue (for latent MTrPs) or red (for active MTrPs) marker. Prior to the start of the study, the researchers agreed upon the MTrPs examination and marking protocols.

In order to establish the prevalence of MPS, patients were considered to suffer from this syndrome if they had at least one active (pain generating) MTrP [3].

The WOMAC is the most widely used instrument to evaluate the symptomatology and function in osteoarthritis of the knee [30]. It contains 24 questions, five about pain (range: from 0 to 20 points), two about stiffness (range: from 0 to 8 points), and 17 about difficulty with physical functions (range: from 0 to 68 points), and can be completed in less than 5 min [31]. An increase in the WOMAC scores (WOMAC pain, WOMAC stiffness, and WOMAC physical function) indicates a degree of deterioration. It has been widely tested in surgical or hospital-based populations and extensively used in clinical trials because of its sensitivity to change and construct validity [31]. The authors of the Spanish version of the WOMAC warn that advanced age of a study population may constitute a possible limitation for its use, which may be relevant for patients undergoing hip or knee replacement surgery as age does not limit the indication for surgery [30].

### 2.5. Data Analysis

To assess comparability of the groups at baseline, we used chi-square test (for categorical variables) and Student's *t*-test (for continuous and ordinal variables). The averages were compared using a Student's *t*-test. If any of the conditions required for its application was not fulfilled (normality according to the Kolmogorov-Smirnov test and homogeneity of the variances, verified using Levene's test), the Mann-Whitney *U* test was used. For the proportions we used Pearson's chi-square test to compare treatment groups and the McNemar test to explore change between time points of study. In order to compare variation rate, Student's *t*-test was used. The Pearson product-moment correlation coefficient was used. To adjust for potential confounding variables, we employed multivariate models (multiple linear regression and multiple logistic regression).

We rejected the one-tailed null hypotheses when the *P* value was lower than 0.05. The data were analysed using the Statistical Package for the Social Sciences 19.0 (SPSS).

## 3. Results and Discussion

No complications related to the dry needling intervention were observed in the T group.

### 3.1. Sample Characteristics

Forty volunteers who were to undergo a total knee replacement procedure participated in the study (29 female and 11 male). The mean (SD) age, height, and weight of the subjects were 72.27 (6.95) years, 1.56 (0.08) m, and 74.75 (10.61) kg, respectively. The rate of women in the whole sample was 70% (55% in the T group and 90% in the S group). The involved knee was the right knee in 60% of subjects (55% in the T group and 65% in the S group). 70% of subjects received general anaesthesia (75% in the T group and 65% in the S group), and in the remaining, 30% spinal anaesthesia was used. The groups were not significantly different (*P* ≥ 0.05) in all characteristics (Table 2) except for gender (*P* = 0.013).

### 3.2. Effect of Dry Needling on VAS Measurements

The initial mean VAS values were higher than the subsequent mean values, which indicates an improvement; this improvement is higher in the T group at the first month, when pain is most severe [23] (see Table 3). 

Since the baseline values of the VAS were higher in the T group, we analysed the variation rate (((value at 1 month − baseline value)/baseline value) × 100). The mean value of the variation rate was higher in the T group (−54.50 (56.60) versus −30.47 (63.23) in the S group), and the difference, analysed with Student's *t*-test, was statistically significant (*P* = 0.048).

A VAS score greater than 40 is considered to represent a significant level of pain [23]. The analysis of this variable (Table 4) showed that before surgery, both groups were similar, although it was slightly higher in the T group. At 1-month follow-up evaluation, the percentage of subjects with a VAS score greater than 40 decreased to 25% from an initial 80% (variation rate = −68.8%). Comparison of baseline values versus 1-month evaluation values of this variable using McNemar test showed that the change was statistically significant in the T group but not in the S group. A comparison of the variation rates of VAS scores greater than 40 between both groups was statistically significant (*P* < 0.05).

**Figure 2:**
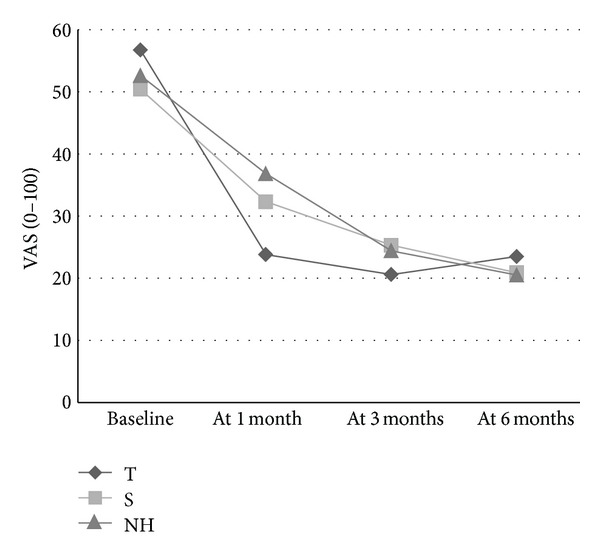
The graph shows average pain scores (VAS) at baseline, and at 1, 3, 6 months in the T group (true dry needling), in the S group (sham dry needling) and in the natural history (NH) [23].

**Figure 3:**
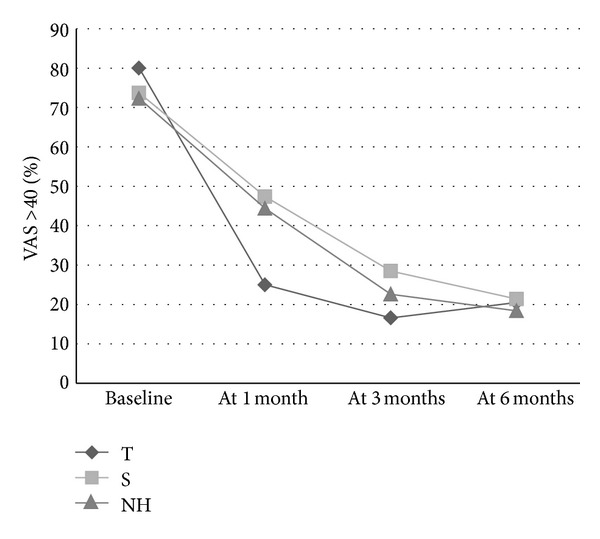
The graph shows percentage of patients with significant pain (VAS > 40) at baseline, and at 1, 3, 6 months in the T group (true dry needling), in the S group (sham dry needling) and in the natural history (NH) [23].

When comparing the outcomes of VAS and VAS > 40 in our study with previously reported results of the natural history of pain after a total knee arthroplasty [23], the results in S group almost completely match those of the results of the natural history (Figures 2 and 3). Figures 2 and 3 also show that subjects in the T group reached the same pain levels in 1-month, as subjects in the S group or subjects with a natural history reached in 6 months.

All subjects had pain before the intervention. In order to find out the percentage of subjects that were pain-free in the different follow-up examinations, variable VAS = 0 was coded and analysed (Table 5). The analysis showed that there was an important difference between both groups at 1-month evaluation, with a significantly higher percentage of pain-free subjects in the T group as compared to the S group.

Since there were statistically significant differences between groups regarding gender, a multivariate analysis was made (a multiple regression for VAS and a logistic regression for VAS > 40 and VAS = 0) to adjust the effect of the intervention on VAS changes by gender. The inclusion of gender in the analysis did not modify the results in any of the variables. Other variables such as age, BMI, type of anesthesia, and baseline values of WOMAC questionnaire were also included in the multivariate model (not shown here) with no changes observed in significance. Therefore, we did not find any variable that was biasing the results of the analysis.

### 3.3. Effect of Dry Needling on Analgesics Requirements

The use of analgesic medication was significantly lower in the T group (31.8%) than in the S group (68.2%) using a chi-Square test (*P* = 0.01).

### 3.4. Correlations between VAS and the Presence of Myofascial Trigger Points

Patients are considered to suffer from MPS if they have at least one active (pain generating) MTrP [3]. In our study, the baseline prevalence of MPS in the whole sample was 75% and decreased much more in the T group (30%) than in the S group (11%), which is nearly a three-fold difference, in the first-month follow-up visit (Table 6). However, despite this difference, the variation rate between baseline and 1-month follow-up visit in both groups was not statistically significant (*P* = 0.06).

MTrPs are persistent sources of peripheral nociceptive inputs, responsible for peripheral [32], and central sensitization [33]. Referred pain from active MTrPs is considered a manifestation of central sensitization [34]. Some studies report a correlation between central sensitization and MTrPs [34, 35] and with knee osteoarthritis [24, 36]. The inactivation of MTrPs and the reduction of referred pain are the results of the desensitizing effects of the treatment. Dry needling causes desensitizing effects in patients with MPS [33, 37], which could account for the observed differences between groups, both in the VAS and in the prevalence of MPS.

### 3.5. Effect of Dry Needling on WOMAC Scores

For all items on the WOMAC, the T group was worse at baseline and throughout all the follow-up checkpoints. Differences between groups were not statistically significant (Table 7). The results of the WOMAC did not correlate with the scores of the VAS, which may be attributed to the difficulty that many subjects experienced with interpreting several test items and completing the WOMAC questionnaires properly. According to Escobar et al., the Spanish version of the WOMAC does have age limitations [30]. They further highlighted that with advanced age, the number of responses to test items and their interpretation may be limited. The mean age of the subjects in the current study was 72.27 (SD = 6.95). In addition, we used the 5-point Likert-type WOMAC questionnaire, instead of the 100 mm visual analog scale format, since, to our knowledge, there was not a validated version of this later format of the WOMAC questionnaire in Spanish. The 100 mm visual analogue scale format has shown a better performance for pain and physical function subscales of the WOMAC questionnaire [38]. These two issues could question the validity of WOMAC results in our sample.

### 3.6. Effect of Dry Needling on Other Measures

No differences between groups were found regarding results in range of motion or strength in any of the follow-up visits.  Table 8 shows these results in the first-month examination. ROM results can be explained by joint limitations due to the arthroplasty and to scar tissue retractions in both capsule and skin. Nevertheless, since MTrPs are considered to limit muscle strength, it could have been expected that the decrease in the number of MTrPs, in the prevalence of MPS, and in pain during the first month would have resulted in an increase in strength that could not be seen in our patients. We only measured the isometric peak value of strength in a single contraction in knee flexion and in knee extension. Further research should employ other outcome measures such as isotonic and endurance measures to evaluate if differences could be detected in this parameter.

### 3.7. Local Twitch Responses under Anaesthesia

In normal conditions, the rapid needle insertion technique employed in the T group usually elicits brief contractions (LTRs) of the taut band that harbours the MTrP [3]. LTRs are considered to be spinal reflexes [39]. Since our subjects were anesthetized, we did not expect to elicit LTRs during the needling and did not plan any data collection on this issue. Nevertheless, to our surprise, LTRs were elicited in some muscles in most of the subjects in the T group in which spinal anaesthesiology was being used (25% of subjects in the T group) and in one muscle (gastrocnemius) in one of the subjects under general anaesthesia. Unfortunately, we did not collect detailed data about this issue. Although it has been reported that elicitation of these contractions usually correlates with better clinical outcomes of dry needling treatments [7], the type of anesthesia employed in our subjects did not seem to affect the results, probably because of the small number of subjects in which this type of anesthesia was used. Irrespective of its influence in our study, the fact that LTRs could be elicited in patients under anesthesia deserves special attention in future research studies as it could mean that local transmission mechanisms could be more important than usually considered [40].

### 3.8. Limitations of the Study

The main drawback of this study is the small sample size, which together with the lack of a prior power calculation may have caused type II errors.

The main objective of our study was to compare the effect of MTrP dry needling versus placebo. Nevertheless, our design does not allow differentiating the effect of MTrP dry needling from the possible neuromodulating effect of the needling itself. Further studies could address this issue using a control group in which needling of the muscle outside the MTrP was applied.

## 4. Conclusions

A single, brief, and safe dry needling treatment applied under anaesthesia in lower limb MTrPs reduced the pain in the first month after total knee replacement surgery, when pain is highest. Dry needling of MTrPs in the lower limb allowed patients to reach the same degree of pain reduction in 1-month as the subjects with a natural history or placebo intervention achieved in 6-months. It significantly decreased the need for postsurgical analgesia. 

This study demonstrates that dry needling is superior to placebo in controlling myofascial pain after a knee arthroplasty. The study introduced a novel placebo methodology for dry needling with a real blinding procedure, which could be utilized in similar studies with different co-morbid conditions, or in studies of myofascial pain concomitant with other surgical conditions of other joints so as to avoid the possible interference of the surgical treatment with the intervention on MTrPs.

Since a single treatment of MTrPs within the context of a knee replacement surgery has proven to be effective in pain reduction after the intervention, it could be conceivable that a more complete treatment program of MTrPs, either before or after the surgery, could be of great help to reduce pain in these patients. Research is needed to test this hypothesis.

## Figures and Tables

**Table 1:** Examination protocol.

	Tensor fasciae latae	Hip adductors	Hamstrings	Quadriceps	Gastrocnemius	Popliteus
Hip position	Extension Lateral rotation	Flexion Abduction Lateral rotation	Flexion Abduction for medial and adduction for lateral muscles	Flexion	Flexion	Flexion Abduction Lateral rotation
Knee position	Extension	Flexion	Flexion	Flexion	Flexion	Flexion

All muscles were examined with the subject in supine position.

**Table 2:** Preintervention groups characteristics (baseline).

	T group (true dry needling) *n* = 20	S group (sham dry needling) *n* = 20	*P* value
Age (years)	71.65 (6.06)	72.90 (7.85)	0.570
Body mass index (Kg/m^2^)	73.57 (11.53)	75.51 (9.33)	0.580
Days hospitalization	8.11 (1.79)	7.58 (2.04)	0.403
VAS (0–100)	56.75 (22.31)	50.37 (16.76)	0.321
WOMAC pain (0–20)	8.10 (2.45)	7.90 (4.60)	0.837
WOMAC stiffness (0–8)	4.05 (1.61)	3.15 (2.16)	0.805
WOMAC function (0–68)	28.48 (8.54)	27.58 (13.50)	0.149
ROM (°)	89.35 (19.191)	93.20 (20.05)	0.539
Strength FLEX (*N*)	20.51 (10.16)	22.00 (5.27)	0.565
Strength EXT (*N*)	24.34 (9.83)	23.42 (7.12)	0.738
MTrPs (number)	12.75 (4.64)	11.75 (3.46)	0.445
MTrPs active (number)	5.15 (4.74)	3.00 (2.83)	0.090

Values are reported as mean (standard deviation). *P* value obtained using Student's *t*-test.

**Table 3:** Initial and subsequent VAS values.

VAS	Baseline	At 1 month	At 3 months	At 6 months
T group				
Mean	56.75	23.80	20.61	23.51
(SD)	(22.31)	(24.86)	(21.49)	(22.50)
*n*	20	20	18	17

S group				
Mean	50.37	32.30	25.31	20.86
(SD)	(16.76)	(25.72)	(20.03)	(18.58)
*n*	19	18	16	14

*P* value	0.320	0.294	0.516	0.725

(SD: standard deviation). *P* value obtained using Student's *t*-test.

**Table 4:** VAS > 40.

Prevalence VAS > 40	Baseline	At 1 month	*P* value	Variation rate
T group	80.0%	25.0%	0.003	−68.8%
	*n* = 20	*n* = 20		

S group	73.7%	47.4%	0.289	−35.7%
	*n* = 19	*n* = 18		

Values are percentages. Variation rate = [(percentage at 1 month − percentage at baseline)/percentage at baseline] ∗ 100. *P* value obtained using the McNemar test.

**Table 5:** VAS = 0.

Prevalence VAS = 0	Baseline	At 1 month	Variation rate	*P* value
T group	0.0%	35.0%	−35.0%	0.042
	*n* = 20	*n* = 20		
S group	0.0%	10.5%	−10.5%	
	*n* = 19	*n* = 18		

Values are percentages. *P* value obtained using Pearson chi-square test. Variation rate was calculated using VAS > 0.

**Table 6:** Prevalence of myofascial pain syndrome.

	Baseline	At 1 month	At 3 months	At 6 months
T group	80%	50%	50%	59%

S group	70%	59%	53%	64%

**Table 7:** WOMAC.

	Baseline	At 1 month	At 3 months	At 6 months
WOMAC pain (0–20)				
T	8.10 (2.44)	5.36 (3.85)	4.50 (3.39)	3.24 (3.03)
S	7.90 (3.59)	4.43 (2.99)	3.26 (2.25)	3.13 (2.72)

WOMAC stiffness (0–8)				
T	4.05 (1.61)	2.26 (1.40)	1.94 (1.69)	1.76 (1.52)
S	3.15 (2.16)	2.17 (1.50)	1.87 (1.78)	1.67 (1.59)

WOMAC function (0–68)				
T	28.48 (8.54)	16.94 (10.68)	13.82 (11.48)	9.70 (7.36)
S	27.58 (13.50)	12.92 (8.29)	10.64 (10.42)	10.53 (11.52)

Values are reported as mean (standard deviation). *P* value obtained using Pearson's chi-square test.

**Table 8:** ROM and strength values at 1-month follow-up examination.

	Group	*n*	Mean	SD	*P* value
ROM	T group	20	74.10	18.80	0.31
	S group	18	77.11	15.31	

Strength FLEX	T group	20	20.49	5.99	0.99
	S group	18	21.25	6.13	

Strength EXT	T group	20	23.01	6.56	0.95
	S group	18	24.11	6.54	

SD: standard deviation. *P* value obtained using Student's *t*-test.
